# Pelvic U-Net: multi-label semantic segmentation of pelvic organs at risk for radiation therapy anal cancer patients using a deeply supervised shuffle attention convolutional neural network

**DOI:** 10.1186/s13014-022-02088-1

**Published:** 2022-06-28

**Authors:** Michael Lempart, Martin P. Nilsson, Jonas Scherman, Christian Jamtheim Gustafsson, Mikael Nilsson, Sara Alkner, Jens Engleson, Gabriel Adrian, Per Munck af Rosenschöld, Lars E. Olsson

**Affiliations:** 1 Radiation Physics, Department of Hematology, Oncology, and Radiation Physics, Skåne University Hospital, Lund, Sweden; 2 Department of Translational Medicine, Medical Radiation Physics, Lund University, Malmö, Sweden; 3 Department of Hematology, Oncology, and Radiation Physics, Skåne University Hospital, Lund, Sweden; 4 Centre for Mathematical Sciences, Lund University, Lund, Sweden; 5 Department of Clinical Sciences, Division of Oncology and Pathology, Lund University, Lund, Sweden; 6 Department of Medical Radiation Physics, Lund University, Lund, Sweden

**Keywords:** Radiation therapy, Semantic segmentation, Deep learning, Anal cancer, Organs at risk

## Abstract

**Background:**

Delineation of organs at risk (OAR) for anal cancer radiation therapy treatment planning is a manual and time-consuming process. Deep learning-based methods can accelerate and partially automate this task. The aim of this study was to develop and evaluate a deep learning model for automated and improved segmentations of OAR in the pelvic region.

**Methods:**

A 3D, deeply supervised U-Net architecture with shuffle attention, referred to as Pelvic U-Net, was trained on 143 computed tomography (CT) volumes, to segment OAR in the pelvic region, such as total bone marrow, rectum, bladder, and bowel structures. Model predictions were evaluated on an independent test dataset (n = 15) using the Dice similarity coefficient (DSC), the 95th percentile of the Hausdorff distance (HD_95_), and the mean surface distance (MSD). In addition, three experienced radiation oncologists rated model predictions on a scale between 1–4 (excellent, good, acceptable, not acceptable). Model performance was also evaluated with respect to segmentation time, by comparing complete manual delineation time against model prediction time without and with manual correction of the predictions. Furthermore, dosimetric implications to treatment plans were evaluated using different dose-volume histogram (DVH) indices.

**Results:**

Without any manual corrections, mean DSC values of 97%, 87% and 94% were found for total bone marrow, rectum, and bladder. Mean DSC values for bowel cavity, all bowel, small bowel, and large bowel were 95%, 91%, 87% and 81%, respectively. Total bone marrow, bladder, and bowel cavity segmentations derived from our model were rated excellent (89%, 93%, 42%), good (9%, 5%, 42%), or acceptable (2%, 2%, 16%) on average. For almost all the evaluated DVH indices, no significant difference between model predictions and manual delineations was found. Delineation time per patient could be reduced from 40 to 12 min, including manual corrections of model predictions, and to 4 min without corrections.

**Conclusions:**

Our Pelvic U-Net led to credible and clinically applicable OAR segmentations and showed improved performance compared to previous studies. Even though manual adjustments were needed for some predicted structures, segmentation time could be reduced by 70% on average. This allows for an accelerated radiation therapy treatment planning workflow for anal cancer patients.

**Supplementary Information:**

The online version contains supplementary material available at 10.1186/s13014-022-02088-1.

## Background

External beam radiation therapy combined with chemotherapy, is the treatment of choice for patients suffering from anal cancer. The overall treatment planning process involves many manual steps, such as treatment plan optimization, as well as manual delineation of organs at risk (OAR) and target volumes. Delineation can be performed based on different image modalities, such as computed tomography (CT), positron emission tomography or magnetic resonance imaging. This manual delineation operation can produce semantic segmentations, where each pixel, or voxel for 3D volumes, is classified as belonging to a set of predefined classes. Nevertheless, manual delineation is a time-consuming process and suffers from inter-observer variability [1–4].

Medical image segmentation has become more automated in the field of radiation therapy (RT). A common, automated method, is atlas-based segmentation (ABS) [5], used in several commercially available software solutions [6]. ABS algorithms use libraries of predefined, expert-delineated structures, that vary in size and shape to cover anatomical variations. These predefined structures can be transferred to a new image with the help of image registration methods using a single atlas or multiple ones. One disadvantage of ABS-based methods is that all data used to generate one or several atlases must be available during matching, making huge atlases not feasible in clinical workflows [7]. As an alternative to ABS, the use of neural networks has recently been considered instead. In fact, current state-of-the-art methods for semantic segmentation in general, predominantly exploit various neural network models. As examples of methods for semantic segmentation, Badrinarayanan et al. [8] explored an encoder-decoder architecture, Ronneberger et al. [9] introduced the U-Net architecture, Wu et al. [10] explored dilated convolutions in a fully connected network and Takikawa et al. [11] utilized gated convolutional layers for a shape stream, aiming to find boundaries of objects. Incorporating mechanisms for attention into neural network models has become an additional important concept towards semantic segmentation that aims to push the state-of-the-art. For example, Chen et al. [12] utilized attention masks to fuse feature maps from different branches, Yuan et al. [13] proposed ways to make use of object context and Huang et al. [14] explored the use of criss-cross attention blocks.

Expanding on ideas from general semantic segmentation, recent proposed neural network models have shown great performance in medical image segmentation tasks, and are now even used for RT applications, such as OAR and target segmentation for head and neck, prostate, and breast cancer patients [15–19]. In addition, neural network models have shown to outperform ABS methods [20, 21] and can reduce the overall manual intervention time [21, 22].

Even though many researchers have incorporated neural network-based segmentation methods for RT purposes, only a few models for automatic segmentation of pelvic OAR and target structures have been proposed [18, 23–25]. Notably, automated deep learning-based segmentation methods have rarely been applied to complex pelvic OAR structures, like small and large bowel, and resulted in relatively unsatisfactory segmentation metrics [23–25]. Furthermore, a detailed evaluation of the segmentation predictions, including both quantitative and qualitative measures, is not always presented, making it difficult to evaluate the full clinical applicability.

The aim of this work was to develop and evaluate a deep learning-based model, for automated and improved multi-label segmentation of ten OAR structures in the pelvic region for anal cancer patients, these being total bone marrow (TBM), lower pelvic bone marrow (LPBM), iliac bone marrow (IBM), lumbosacral bone marrow (LSBM), bowel cavity, all bowel, small bowel, large bowel, rectum, and bladder. Mainly exploiting and combining ideas from U-Net [9], deep supervision [26] and Shuffle Attention (SA) [27], we present a modified U-Net architecture, using depth-weighted deep supervision and SA blocks [27], combining the advantages of spatial and channel-wise attention mechanisms [28]. In this work, we refer to our modified U-Net as Pelvic U-Net. Segmentation performance was evaluated using quantitative, observational, dosimetric and time-based measures.

## Methods

### Dataset

The dataset used in this study consisted of 169 consecutive patients with squamous cell carcinoma of the anus (anal cancer) treated with RT at Skåne University Hospital, Lund, Sweden, during the period Aug. 2009–Dec. 2017. CT image acquisition was performed on four different CT scanners (Siemens SOMATOM (Siemens AG, Munich, Germany), Philips GEMINI TF (Philips, Eindhoven, Netherlands), GE Discovery 690 and GE HiSpeed NX/I (General Electric, Boston, USA)), with 120 peak kilovoltage (pKV) and standard vendor-specific, dose-sparing, tube current settings. OAR structures were retrospectively delineated on each slice of the RT planning CT (slice thickness 3 or 2.5 mm) by two clinical experts (MPN, JS), as previously described [29]. In brief, the outer borders of the bladder and the rectum were contoured. The inferior limit of the rectum was defined as the inferior border of the ischial tuberosities (therefore, parts of the anal canal were sometimes included), and the superior border was the rectosigmoid junction. Bowel cavity was delineated according to the definition used by Devisetty et al. [30], as an envelope from the anterior abdominal wall to the most posterior extent of the bowel, and from bowel edge to bowel edge in the lateral direction. The inferior limit of the bowel cavity was the rectosigmoid junction or the most caudal extent of small/large bowel, whichever was most inferior. For small and large bowel, the outer border of individual bowel loops was contoured. The bowel structure, referred to as ‘all bowel’ in this work, was a summation of small and large bowel. In cases where small bowel was difficult to separate from large bowel on the non-contrast enhanced planning CT, previous diagnostic CT volumes with oral contrast were reviewed. Pelvic TBM was contoured in accordance with Mell et al. [31] as the external contour of bones from the superior border of the L5 vertebral body to the inferior border of the ischial tuberosities. To assist in the manual delineation process of bone marrow structures, a thresholding algorithm in the Eclipse treatment planning system (TPS) (Version 15.6, Varian Medical Systems, Palo Alto, CA, USA) was used. Pelvic sub volumes (LPBM, IBM, and LSBM) were also contoured in accordance with Mell et al. [31]. Expert delineations are subsequently referred to as the ground truth (GT) data.

Eleven patients were excluded from the dataset due to the occurrence of hip prosthesis. From the remaining 158 patients, 143 were used to train the Pelvic U-Net, while 15 patients were used as an independent test set, to evaluate the performance of the final model. The test patients were never used for model training or validation. All patients were exported from the Eclipse TPS using the Eclipse Scripting Application Programming Interface (ESAPI), Version 15.6, Varian Medical Systems, Palo Alto, CA, USA.

The study was approved by the Regional Ethics Board of Lund, Sweden (EPN Lund, Dnr 2013/742).

### Pre- and post-processing for model training and testing

CT images and the corresponding Digital Imaging and Communications in Medicine (DICOM) RT structure sets were extracted from the TPS. Annotated structures were saved as binary segmentation masks and combined into a 3D volume with 10 separate image channels, one channel for each structure. The segmentation task was treated as a multi-label problem, where classes are mutually non-exclusive. Masks and images were cropped around the body contour and resampled to a common voxel size of $3.0 \times 1.0 \times 1.0$ mm (inferior-superior, right-left, anterior–posterior directions) using nearest neighbor and bi-cubic interpolation, respectively. CT intensity values were normalized using global z-score normalization, by computing the mean and standard deviation of all foreground voxel values over the entire training dataset. Finally, CT intensity values were clipped to the 95th and 5th percentile.

As the GT delineation boundary of the bowel structures was defined 2 cm superior to the planning target volume (PTV), which had a large variation in size among the patients, a uniform segmentation boundary could not be provided to the model. Therefore, voxels inside the patient’s body, but outside the proximate boundary of the GT were excluded from the loss calculation during training.

Segmentations predicted by our suggested model were resampled and zero-padded to the same size as the original, corresponding CT image volumes. For evaluation purposes, bowel structures were clipped superior to their corresponding GT delineation. Finally, a new DICOM RT structure set was generated [32] and imported to the TPS. All pre- and post-processing operations were performed using in-house developed Python scripts.

### CNN architectures

**Fig. 1:**
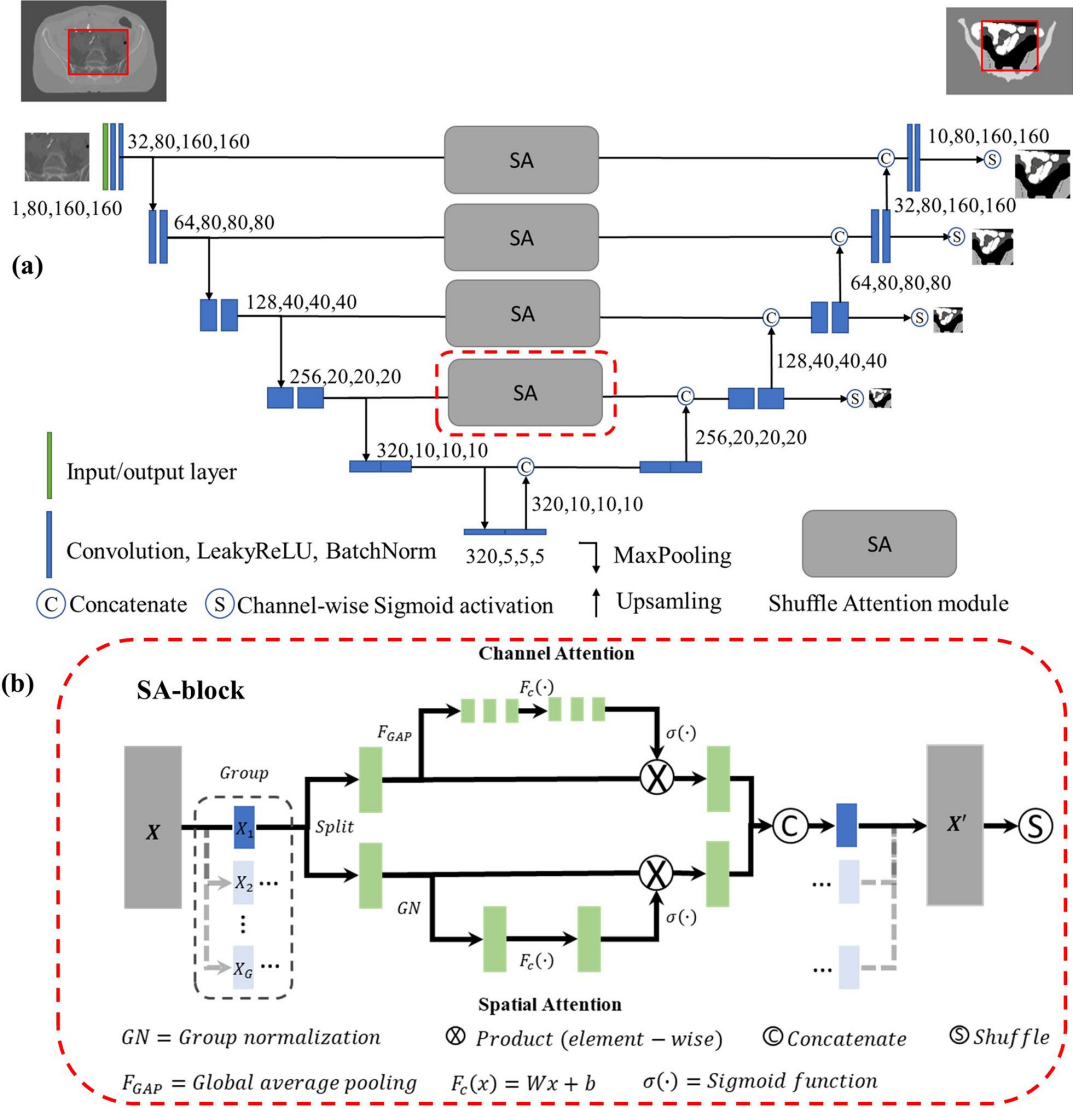
Proposed, deeply supervised Pelvic U-Net architecture for organs at risk (OAR) segmentation in the pelvic region (**a**). 3D patches with a size of $1 \times 80 \times 160 \times 160$ pixels are extracted from computed tomography (CT) image volumes and used as the encoder input. A series of convolutional and max pooling operations is then applied to the input patch for feature extraction purposes. Feature map upscaling in the decoder part is performed using trilinear interpolation. High level features from the encoder are copied and concatenated with low level features using skip connections. In addition, shuffle attention (SA) blocks are incorporated into the skip connections, combining spatial and channel attention (**b**)

As a backbone model we used a modified U-Net architecture, first introduced by Ronneberger et al. [9]. The U-Net model consists of a contraction (encoder) part and a localization (decoder) part. Input patches with a size of $1 \times 80 \times 160 \times 160 \left( {c \times d \times h \times w} \right)$ pixels, where c is the number of image channels, d the depth of the patch volume, w the width and h the patch height, were used as the input to the convolutional neural network (CNN) (Fig. 1a).

The contracting part of the U-Net is used to extract image feature representations at multiple levels and consists of a series of convolutional blocks. Each convolutional block consists of convolutional layers with a 3 × 3 convolutional kernel followed by Batch Normalization [33] with a constant for numerical stability of $\varepsilon = 1.00 \times 10^{ - 5}$, as well as leaky rectified linear unit (Leaky ReLU) activation with a negative slope of 0.01. Each convolutional block is followed by a MaxPooling layer with a 2 × 2 kernel and a stride of two, for downsampling purposes. The localization path projects the low resolution, discriminative features into a higher resolution pixel space using trilinear upsampling operations, where each upsampling is followed by a convolutional block. In addition, the U-Net architecture uses skip connections at multiple levels, to copy features from the contracting path and concatenate them with the corresponding features of the localization path. This helps to recover information lost by the downsampling operations performed in the encoder part of the model [34].

In our suggested Pelvic U-Net, deep supervision, introduced by Lee et al. [26], was added. Deep supervision uses secondary segmentation maps of different resolutions, derived from the Pelvic U-Net architecture. These are used to compute weighted auxiliary losses, which are added to the main loss function. Deep supervision has successfully been used in other medical imaging segmentation tasks, demonstrating faster network convergence [35–38].

Finally, inspired by the work of Zhang and Yang [27], we incorporated shuffle attention (SA) modules into our model (Fig. 1b). The SA module combines the advantages of spatial attention and channel-wise attention, focusing on what features are important, and where these features can be found. A SA module first divides a feature map $X$ into $G$ groups, where each group represents sub-features of $X$. The number of groups is a hyperparameter, which was chosen to be equal to $G = 16$ in our work. Each sub-feature is further split into a channel attention and a spatial attention branch. The channel attention branch uses global average pooling (GAP) [39] followed by a sigmoid activated attention mechanism. The spatial attention block uses group normalization (GN) followed by sigmoid activation. The outputs of the two attention branches are then combined by concatenation. Finally, a channel shuffle mechanism introduced in ShuffleNet V2 [40] is used, to enable cross information flow across the channel dimension. A PyTorch (Facebook, CA, USA) implementation of the Pelvic U-Net can be found on our GitHub repository https://github.com/MLRadfys/Multilabel-semantic-segmentation-for-pelvic-OAR-structures.git.

### Model training

Model training was performed by 5-fold cross-validation using the adaptive moment estimation (Adam) optimizer [41], a learning rate of $lr = 0.01$ and a batch size of 2. The learning rate was decreased using a polynomial learning rate decay schedule with a power of 0.9. Each cross-validation model was trained for a total of 1000 epochs, with 250 iterations per epoch. 3D patches with a size of $1 \times 80 \times 160 \times 160$ pixels $\left( {c \times d \times w \times h} \right)$ were randomly cropped from CT image volumes and fed into the input layer of the CNN. To avoid overfitting during training, data augmentation techniques in form of random rotations (± 15°, around inferior-superior axis), mirroring (inferior–superior, left–right axis), scaling (0.85–1.25) and gamma augmentations (0.7–1.5) were used. All spatial augmentations were performed in 2D and implemented using the batchgenerators Python package [42]. Model optimization was performed by combining the soft Dice and the binary Cross-entropy (BCE) loss [43]:

1 $$\begin{aligned} & {\mathcal{L}} = {\mathcal{L}}_{SoftDice} + {\mathcal{L}}_{BCE} \\ & {\mathcal{L}}_{SoftDice} = - \frac{{2\mathop \sum \nolimits_{i = 1}^{N} \hat{y}_{i} y_{i} + \varepsilon }}{{\mathop \sum \nolimits_{i = 1}^{N} \hat{y}_{i} \mathop \sum \nolimits_{i = 1}^{N} y_{i} + \varepsilon }} \\ & {\mathcal{L}}_{BCE} = - \mathop \sum \limits_{i = 1}^{N} y_{i} \log \left( {\hat{y}_{i} + \varepsilon } \right) + \left( {1 - y_{i} } \right)\log \left( {1 - \hat{y}_{i} + \varepsilon } \right), \\ \end{aligned}$$

where $y_{i}$ represents an annotated GT voxel, $\hat{y}_{i}$ the corresponding prediction and N the total number of voxels. A small constant $\varepsilon = 1 \times 10^{ - 8}$ is added to both the soft Dice loss and the BCE loss to ensure numerical stability. Segmentation maps obtained at the different deep supervision stages in the decoder path of the model (see Sect. 2.3), were weighted exponentially in the loss function, where the lowest weight was assigned to the decoder output with the lowest resolution, and the highest weight was assigned to the decoder output with the highest resolution [38]:

2 $$w = \mathop \sum \limits_{i = 1}^{S - 1} \frac{1}{{2^{i} }} ,$$

where $S$ is the number of Pelvic U-Net stages. All weights were normalized to sum up to one. The final loss, excluding the lowest stage of the Pelvic U-Net, is given by:

3 $${\mathcal{L}}_{total} = \mathop \sum \limits_{i = 1}^{N} w_{i} \left[ {{\mathcal{L}}_{SoftDice} + {\mathcal{L}}_{BCE } } \right]_{i}$$

To reduce load on the graphics processing unit and to accelerate model training, automatic mixed precision was used. Model training was performed on a single GeForce RTX 2080 TI graphic cards (NVIDIA, USA), with a training time of five days per fold. The final model was established by averaging the output prediction logits of all cross-validation models, resulting in a model ensemble. A detailed evaluation of the different cross-validation folds is provided in the supplementary material (Additional file 1: Table S1-S2, Fig. E1).

### Segmentation evaluation

Segmentations predicted by the Pelvic U-Net, were evaluated using different measures (further described below): 1) quantitative segmentation metrics 2) an observational assessment, 3) a time-based evaluation, comparing our deep learning method to both manual contouring time, and manual correction time of segmentations generated by our model, and by 4) comparing the dosimetric difference between manually delineated contours and the model’s predictions.

#### Quantitative evaluation metrics

To evaluate the segmentation performance of our model, the Dice similarity coefficient (DSC) [44] (Eq. 4), the 95th percentile of the Hausdorff distance (HD_95_) [45] given in mm (Eq. 5), as well as the mean surface distance (MSD) [7] (Eq. 6) in mm were used. The DSC is a measure of overlap between two structures and is given by:

4 $$DSC\left( {A,B} \right) = \frac{{2 \left| {A \cap B} \right|}}{\left| A \right| + \left| B \right|} ,$$

where $\left| A \right|$ and $\left| B \right|$ are the number of voxels in the two volumes $A$ and $B$ and $A \cap B$ is the intersection of both sets, defining the overlapping voxels.

The symmetric Hausdorff distance measures the distance between two surfaces in mm and is given by:

5 $$\begin{aligned} & HD_{K} \left( {A,B} \right) = max\left( {d_{K} \left( {A,B} \right), d_{K} \left( {B,A} \right)} \right) , with \\ & d_{K} \left( {A,B} \right) = K_{a \in A}^{th} \left( {\mathop {\min }\limits_{b \in B} d\left( {a,b} \right)} \right), \\ \end{aligned}$$

where $a$ and $b$ are points of sets $A$ and $B$ and $K_{a \in A}^{th}$ is the Kth percentile of the ordered Euclidean distance norm over $a \in { }A$. In this study, we computed the $K = 95^{th}$ percentile of the Hausdorff distance, which gives a more robust estimate of the maximum error by avoiding outliers [45, 46].

The DeepMind open-source library for surface metrics [47] was used to compute the MSD, which measures the mean distances between two surfaces. The MSD is given by:

6 $$MSD\left( {A,B} \right) = \frac{{\left( {\overline{d}\left( {A,B} \right) + \overline{d}\left( {B,A} \right)} \right)}}{2} ,$$

where $\overline{d}\left( {A,B} \right)$ and $\overline{d}\left( {B,A} \right)$ contain the average minimum distances from points on surface $A$ to points on surface $B$ and vice versa. In our work, we used the MSD as a symmetric measure, by summing the distances and computing their mean value.

#### Observational visual evaluation

**Fig. 2:**
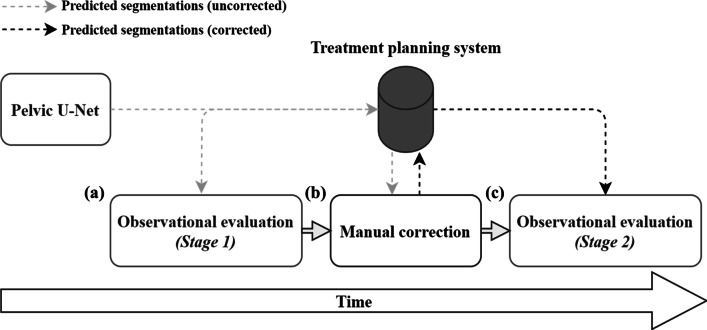
2-stage observational evaluation process of segmentation predictions derived for n = 15 test patients. Uncorrected segmentations were rated on a scale between 1–4 by three radiation oncologists (**a**). Once rated, all structures were manually corrected (**b**) and rated a second time (**c**)

Model predictions for TBM, all bowel, bowel cavity, small bowel, large bowel, and bladder were evaluated in form of a 2-stage, observational, visual assessment, individually performed by three radiation oncologists (JE, SA, GA), with 7–20 years of clinical experience. During the first stage, model predictions for $n = 15$ patients were presented to each of the radiation oncologists (Fig. 2a). All structures were then rated on a scale between 1–4: 1—*Excellent*; 2—*Good*; 3—*Acceptable*; 4—*Not acceptable*.

For an *excellent* segmentation, no or almost no modifications should be needed, while a *good segmentation* requires changes to a limited number of CT slices. An *acceptable* segmentation can still be used but requires corrections in several slices. The used rating method was first presented in the work of Huyskens et al. [48]. In the present study, the number of slices that need to be corrected was set to $n < 10$ slices for good segmentations. Very small deviations $< 5$ mm were not counted. A segmentation was rated as acceptable if $n \ge 10$ slices had to be corrected. A *not acceptable* segmentation was considered useless and would have to be re-delineated for clinical uses.

Once the initial rating of the structures was completed, model predictions were corrected manually for all test patients by one of the radiation oncologists who generated the GT data (MPN, Fig. 2b). A second rating of the manually corrected segmentations was then performed, approximately one month later, to verify that all segmentations were in full agreement with the clinical standard (Fig. 2c). The second rating followed the same procedure as described above.

#### Evaluation of segmentation time

The time needed to manually correct predicted segmentations for the test data was measured per patient and per structure, except for small bowel, large bowel and all bowel, which were corrected simultaneously. All manual corrections where performed by the same radiation oncologist (MN), who generated the GT delineations. Correction time was then compared to the estimated, manual delineation time.

#### Dosimetric evaluation

Dose distributions derived from the clinical treatment plans, optimized based on the manually delineated structures, were overlayed on the predicted segmentations to evaluate any dosimetric differences A new dose optimization was not performed. Structure volume, as well as different DVH inidices (V10_Gy_, V20_Gy_, V30_Gy_, V40_Gy_, V50_Gy_, D_mean_) where extracted from the TPS using ESAPI. Statistical comparison between the DVH indicies for the two structure sets was performed using a non-parametric, two-sided Wilcoxon signed rank test, with a significance level of $\alpha = 0.05$.

## Results

### Quantitative evaluation

**Fig. 3:**
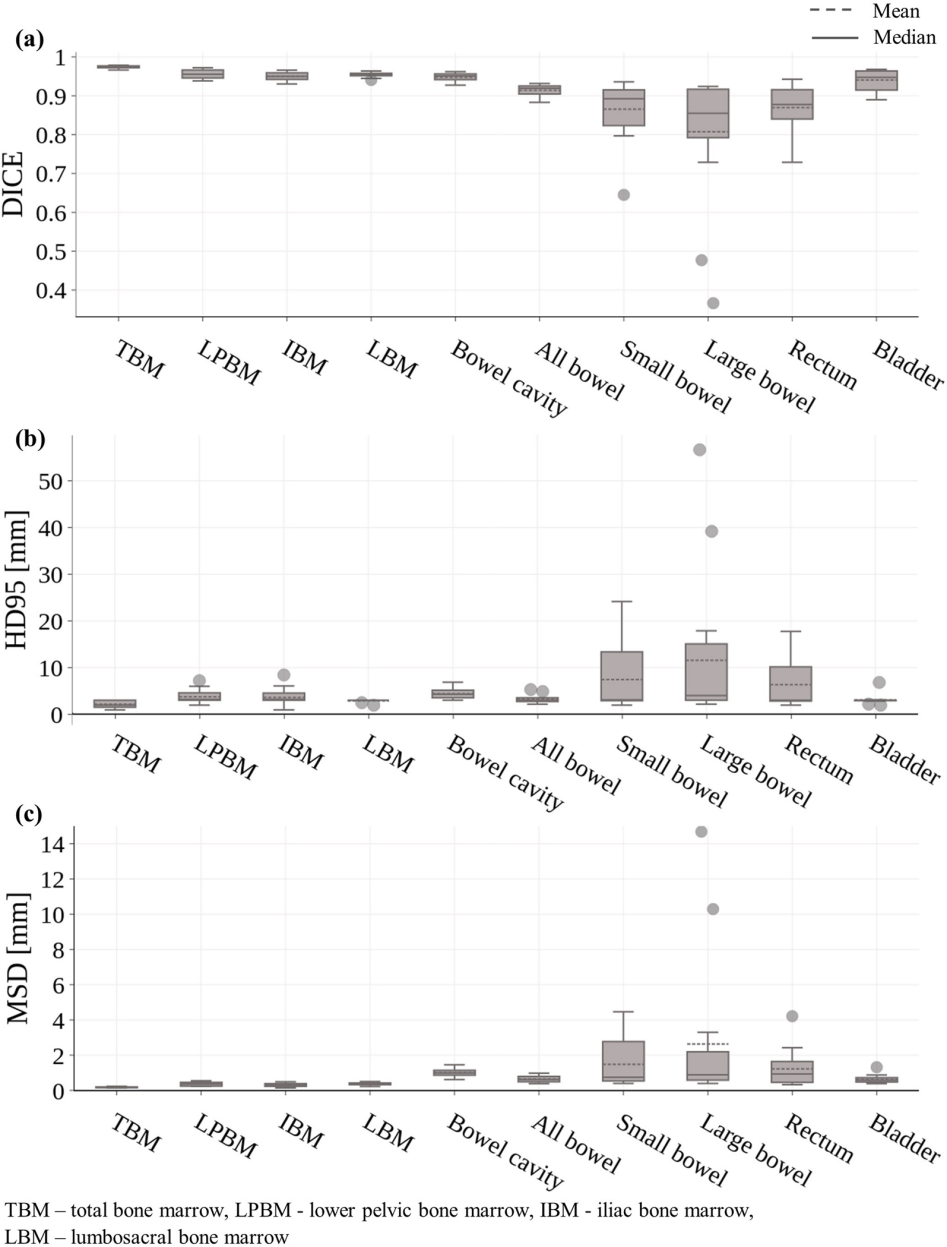
Box and Whisker plot showing the result of the quantitative segmentation evaluation for the test dataset ($n = 15$). ﻿(**a**) Dice similarity coefficient (DSC), (**b**) the 95th percentile of the Hausdorff distance (HD_95_) and (**c**) the mean surface distance (MSD), were computed as the mean value over the test data. Overall, all segmented structures led to high DSC values, while some outliers were observed for small and large bowel structures

Segmentation performance was evaluated using the DSC, HD_95_ as well as the MSD (Table 1 and Fig. 3). All quantitative metrics were calculated as the mean over all 15 test patients and computed on segmentations directly derived from the Pelvic U-Net (without manual corrections).

**Table 1:** Summary of the quantitative evaluation. Dice similarity coefficient (DSC), 95th percentile of the Hausdorff distance (HD_95_), and mean surface distance (MSD) were computed as the mean value over the test dataset (${\text{n}} = 15$)

Quantitative metrics $\left( {n = 15} \right)$			
Structure	DSC $\left( {\overline{x} \pm sd} \right)$	HD_95_ [mm]$\left( {\overline{x} \pm sd} \right)$	MSD [mm] $\left( {\overline{x} \pm sd} \right)$
TBM	0.97 ± 0.00	2.16 ± 0.76	0.18 ± 0.03
LPBM	0.96 ± 0.01	3.77 ± 1.51	0.36 ± 0.11
IBM	0.95 ± 0.01	3.64 ± 1.90	0.30 ± 0.10
LBM	0.95 ± 0.01	2.90 ± 0.29	0.37 ± 0.07
Bowel cavity	0.95 ± 0.01	4.44 ± 1.18	1.01 ± 0.24
All bowel	0.91 ± 0.01	3.22 ± 0.86	0.65 ± 0.19
Small bowel	0.87 ± 0.08	7.64 ± 7.10	1.48 ± 1.33
Large bowel	0.81 ± 0.17	11.51 ± 15.92	2.63 ± 4.16
Rectum	0.87 ± 0.06	6.34 ± 5.20	1.23 ± 1.04
Bladder	0.94 ± 0.03	3.11 ± 1.08	0.63 ± 0.24

*TBM* total bone marrow, *LPBM* lower pelvic bone marrow, *IBM* iliac bone marrow, *LBM* lumbosacral bone marrow, $\overline{x}$ mean, $sd$ standard deviation

Mean DSC values were $> 95\%$ for all different bone marrow structures, with mean HD_95_ values in the range of 2.16–3.77 mm and mean MSD values between 0.18–0.37 mm. For bowel cavity, all bowel, and bladder, mean DSC values were 0.95, 0.91, 0.94, with mean HD_95_ values of 4.44, 3.22 and 3.11 mm, and mean MSD values of 1.01, 0.65 and 0.63 mm, respectively. The predicted segmentations of small bowel and rectum resulted in mean DSC values of 0.87 for both structures, mean HD_95_ of 7.64 and 6.34 mm and mean MSD values of 1.48 and 1.23 mm, respectively. The lowest mean DSC of 0.81 was found for the large bowel structure, with mean HD_95_ and MSD values of 11.51 mm and 2.63 mm, respectively.

A detailed evaluation of the 5-fold cross-validation training can be found in the supplementary section (Additional file 1).

**Fig. 4:**
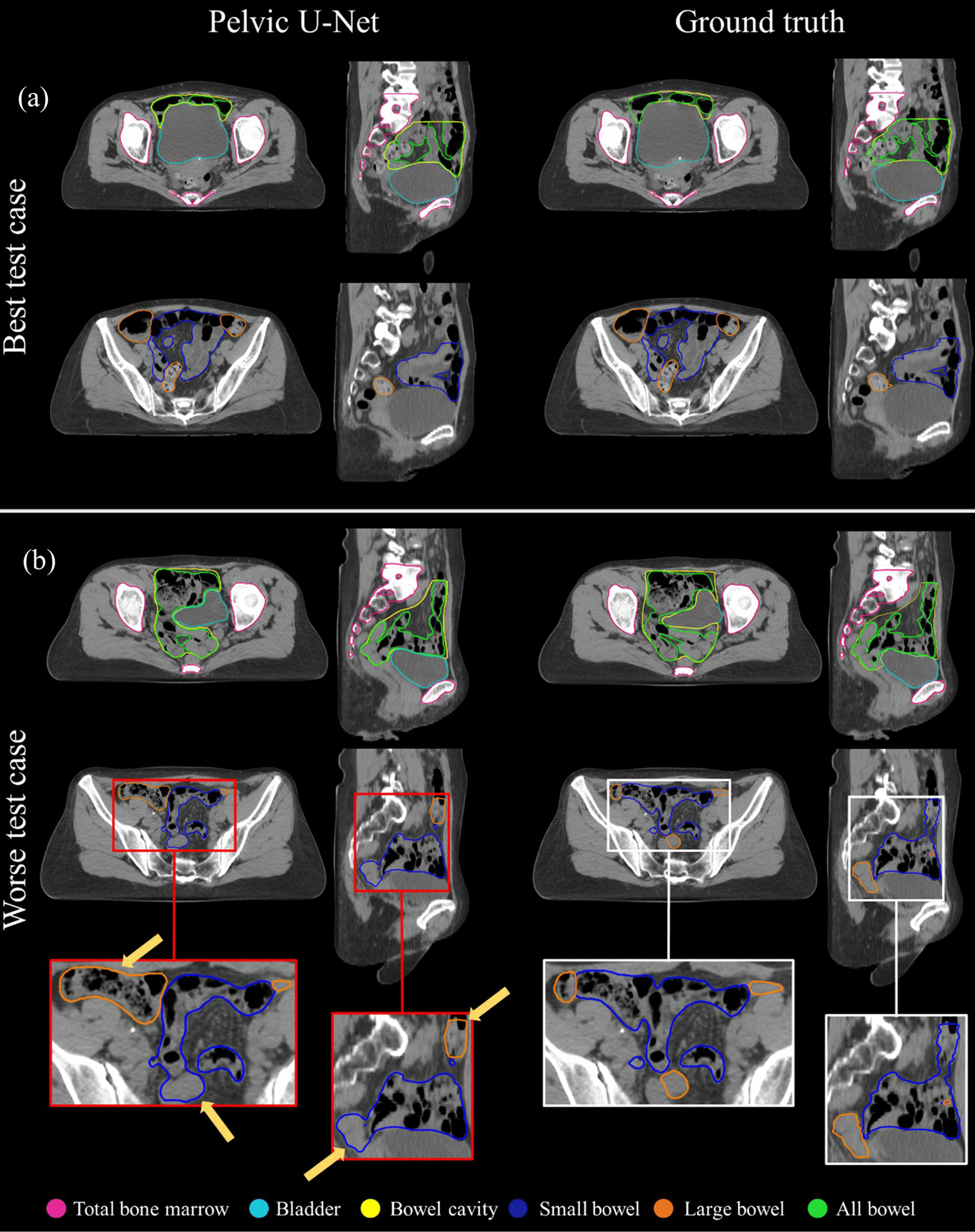
The best (**a**) and the worse (**b**) segmentation result shown for total bone marrow, bladder, bowel cavity, small bowel, large bowel, and all bowel structures. Test cases were chosen based on the minimum and maximum average Dice similarity coefficient (DSC). All presented structures were in good agreement with the ground truth (GT, expert delineation) for the best test case, resulting in a mean DSC of 0.94. For the worst case, a mean DSC of 0.8 was found, mainly due to inaccurate segmentations of the small and large bowel structures, indicated by the yellow arrows. Parts of the small bowel were wrongly classified as parts of the large bowel and vice versa

Figure 4 shows segmentation examples for the best and the worst segmentation case in the axial and sagittal plane. The two patients were chosen according to the minimum and maximum mean DSC value computed over all evaluated structures.

For the best test case, all predicted segmentations were in good agreement with the clinical GT segmentations resulting in a mean DSC of 0.94. For the worst test case, parts of the large bowel were wrongly classified as small bowel and vice versa, which resulted in decreased DSC values for both structures (0.65, 0.37 for small and large bowel, respectively).

**Fig. 5:**
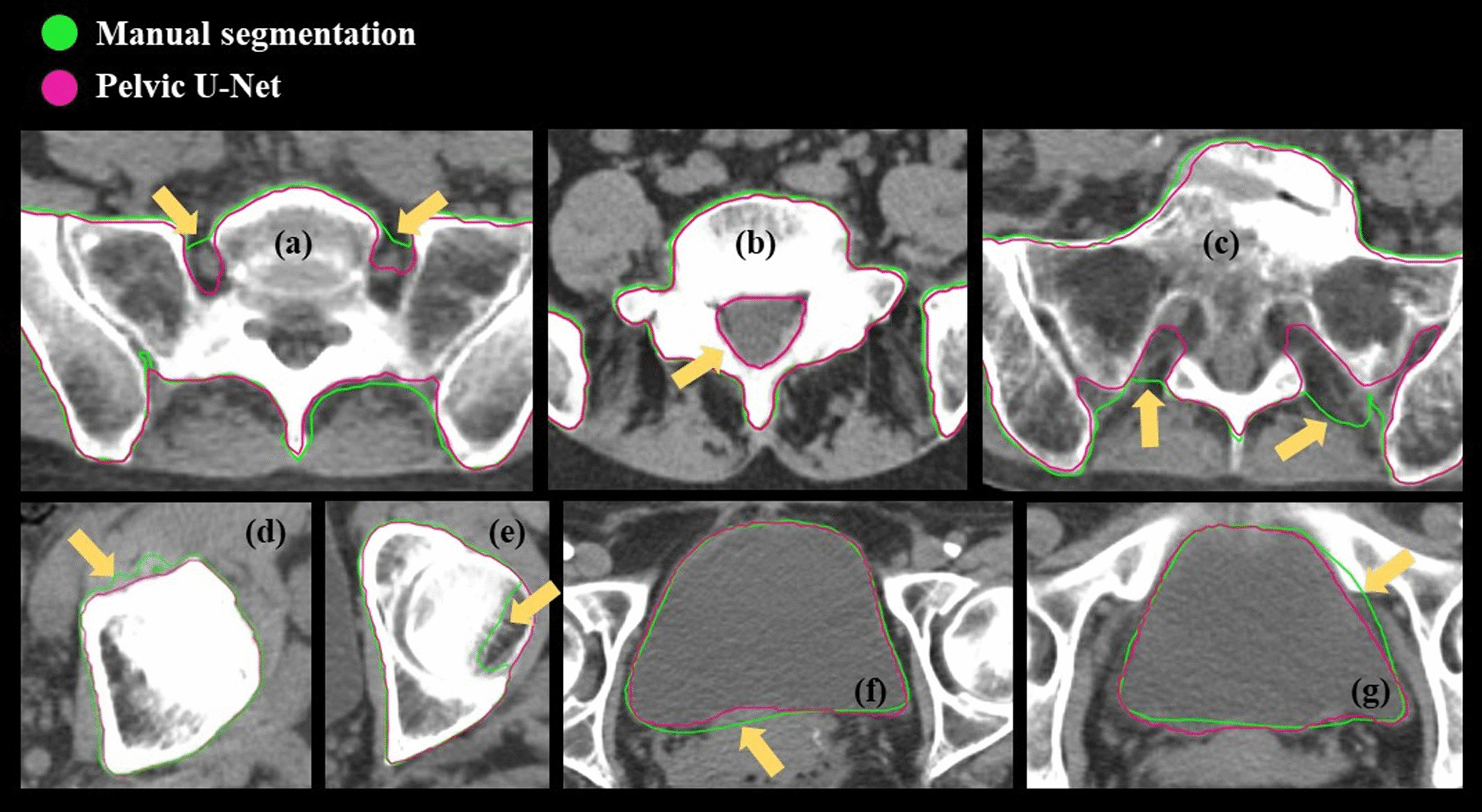
Observations made during a more detailed, visual comparison, between the segmentation structures predicted by the Pelvic U-Net and the manual, ground truth (GT), delineations for multiple test patients. In some 2D image slices, tissue near the actual bone structures was not segmented (**a–c**). Furthermore, incorrect delineations caused by the automatic, thresholding-based segmentation algorithm used for generating the GT bone marrow data were found (**d**, **e**). In addition, rare, manual delineation errors for e.g., the bladder could be observed (**f**, **g**)

Determined through visual inspection of predictions for the test data, tissue near or enclosed by bone structure, usually delineated in the GT data, was not classified as bone by the Pelvic U-Net in occasional CT slices (Fig. 5a–c). In some cases, artefacts, caused by the thresholding algorithm used to assist the radiation oncologists in GT bone segmentations, were found (Fig. 5d, e). In other cases, such as the bladder, differences between model predictions and manual segmentations were observed, where the manual structure was not perfectly delineated (Fig. 5f, g). These findings are elaborated on in more detail in the discussion section of this work.

### Observational evaluation

**Fig. 6:**
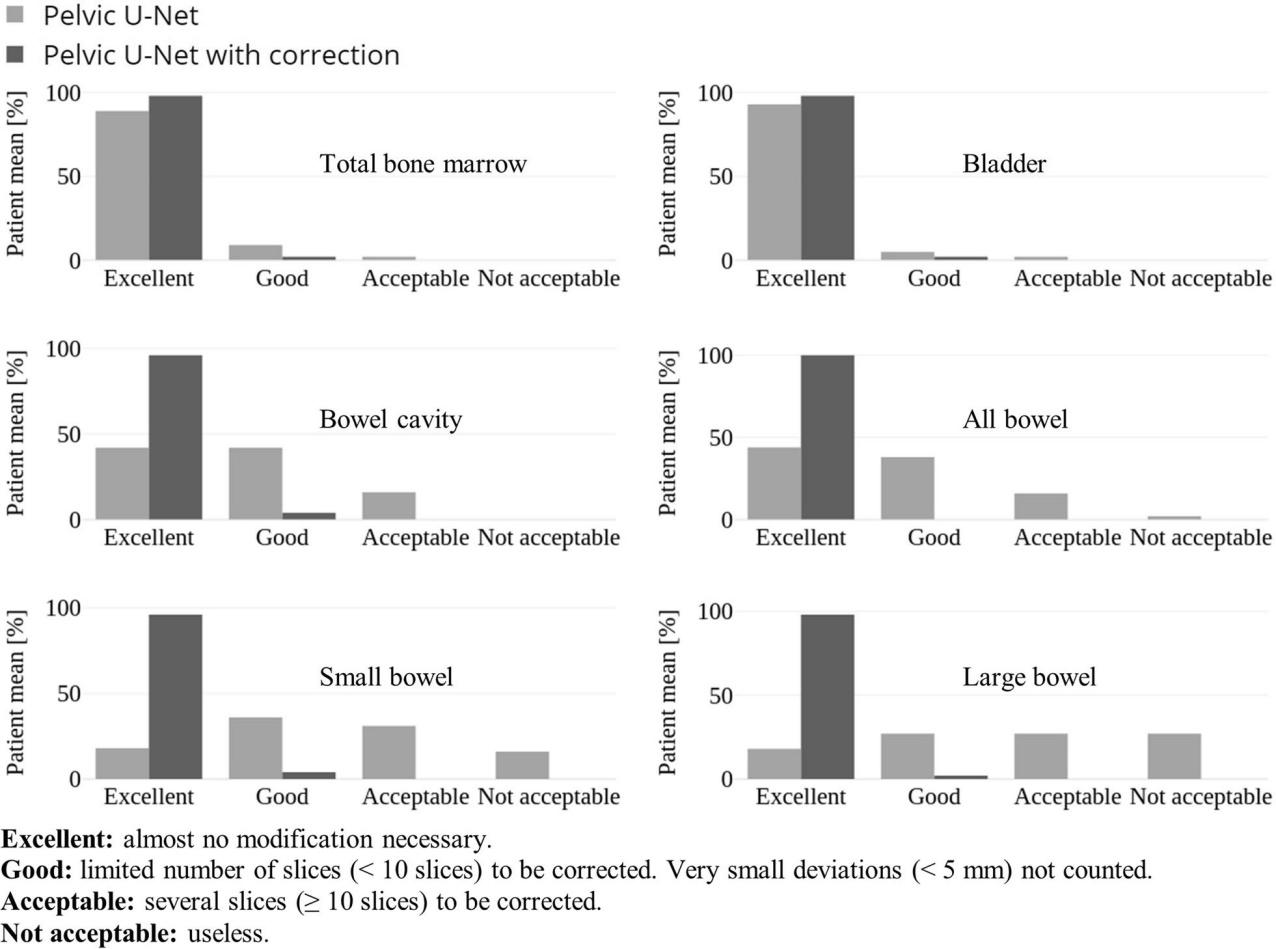
Observational evaluation results of pelvic organs at risk (OAR) segmentations. Segmentation predictions for ${\text{n}} = 15$ test patients were inspected by three clinical experts and rated on a scale between 1–4 (Excellent—Not acceptable). Results are presented as the mean ratings for each structure for both the uncorrected segmentations (stage 1) and the manually corrected segmentations (stage 2)

The result of the 2-stage observational evaluation performed by three clinical experts is summarized in Fig. 6.

Without corrections, TBM and bladder segmentations resulted in an excellent segmentation quality (89% and 93% on average). For bowel cavity, most segmentations were judged to be either excellent (42%) or good (42%), with only minor segmentation faults. For all the above-mentioned structures, none of the model predictions was assessed as non-acceptable. Most all bowel segmentations were assessed as excellent (44%) or good (38%), while only a small fraction (2%) was not acceptable. For small and large bowel, segmentation assessments varied between no modifications needed (excellent) and acceptable (some modifications needed), while 16% of the small and 27% of the large bowel segmentations were found to be non-acceptable and would have to be re-delineated.

After manual correction of the predicted segmentations, structures of nearly all test patients were assessed as excellent (97%), where no modifications are needed, and only a few as good (3%) (evaluation stage 2).

Observer ratings for TBM, bladder and bowel cavity resulted in the same median values, indicating a good agreement between the observes. Small and large bowel structures resulted in median values between 2–3, showing minor variations between the ﻿observes (Table 2).

**Table 2:** Comparison of median ratings between the three observers for all evaluated structures. The same median ratings could be found for total bone marrow (TBM), bladder and bowel cavity. Median ratings for small and large bowel structures varied between 2–3

Observer median ratings ($n = 15$)			
Observer	SA	GA	JE
Structure	Median (range)	Median (range)	Median (range)
TBM	1 (1–2)	1 (1–1)	1 (1–3)
Bladder	1 (1–2)	1 (1–3)	1 (1–1)
Bowel cavity	2 (1–2)	2 (1–3)	2 (1–3)
All bowel	2 (1–3)	2 (1–3)	1 (1–4)
Small bowel	3 (1–3)	2 (1–4)	3 (1–4)
Large bowel	3 (1–4)	3 (1–4)	2 (1–4)

### Evaluation of segmentation time

**Fig. 7:**
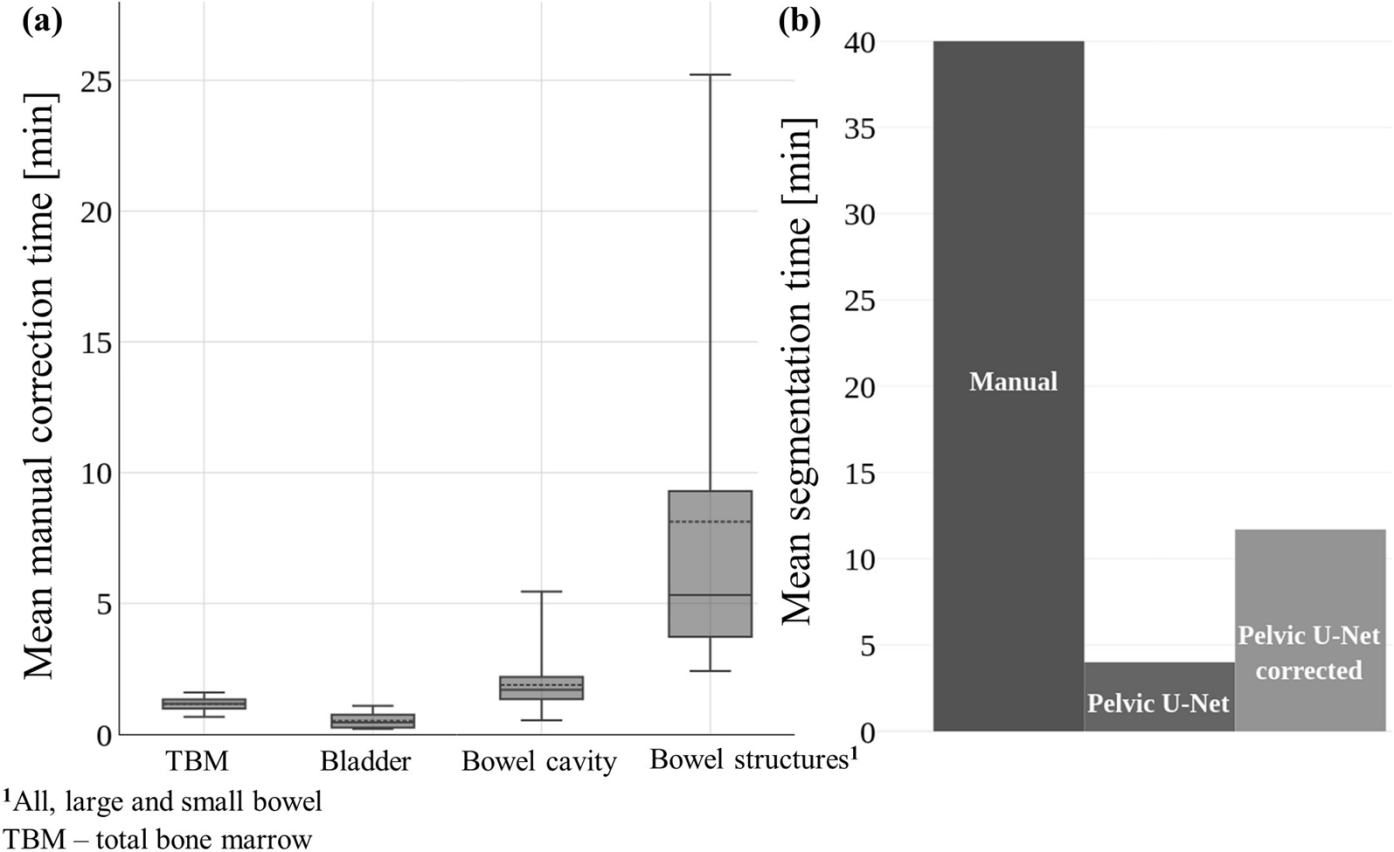
Time-based evaluation result comparing the mean manual delineation time for all test patients (${\text{n}} = 15$) against the time needed to manually correct model predictions and model prediction time (without corrections). Mean manual correction times per structure are shown in the Box and Whisker plot (**a**). Mean segmentation time could be reduced from 40 min to about 12 min per patient using manually corrected model predictions, and to 4 min, using predictions of the Pelvic U-Net only (**b**)

Mean correction times for TBM, bladder, bowel cavity and bowel structures were measured to be 1 min, 1 min, 2 min and 8 min, respectively, resulting in a mean total correction time of 12 min per patient (Fig. 7a). Two clinical experts who generated the manual delineations (MPN, JS), estimated the mean time for complete manual segmentation, e.g., segmenting the evaluated OAR structures from scratch, to be approximately 40 min per patient. With that, our deep learning-based approach, results in a large timesaving of 70% (Fig. 7b). In addition, model prediction time for all OAR structures was measured to be 4 min. This reduces the mean segmentation time by 90%, for cases where no modifications are needed.

### DVH-based evaluation

For bowel cavity, large bowel and small bowel, no significant difference could be found between the dose using the manually, clinical delineations and the model’s predictions (Table 3). A significant difference was found for the DVH indices of the TBM (Volume, V50_Gy_) all bowel (Volume, V10_Gy_, V20_Gy_) and bladder (V10_Gy_, V20_Gy_, V30_Gy_) structures.

**Table 3:** Result of the dosimetric evaluation between the clinical treatment plans, optimized based on the manually delineated structures and the same dose distribution overlayed on the uncorrected segmentations derived from the Pelvic U-Net

*p*-values for evaluated pelvic OAR structures ($\alpha = 0.05$)							
	DVH indices^a^						
Structure	Volume	V10_Gy_	V20_Gy_	V30_Gy_	V40_Gy_	V50_Gy_	Dose_mean_
TBM	** < 0.001**	0.670	0.064	0.285	0.421	** < 0.001**	0.583
Bladder	0.073	**0.041**	**0.035**	**0.026**	0.277	0.715	0.421
Bowel cavity	0.073	0.064	0.073	0.169	0.421	0.557	0.679
All bowel	**0.007**	**0.010**	**0.026**	0.055	0.095	0.175	0.561
Small bowel	0.229	0.268	0.241	0.390	0.583	0.765	0.160
Large bowel	0.073	0.107	0.107	0.229	0.208	0.160	0.151

Bold values indicate statistically significant differences from a two-sided Wilcoxon signed rank test with a significance level of ${\upalpha } = 0.05$.

*OAR* organs at risk, *DVH* dose-volume histogram, *TBM* total bone marrow

^a^Volume [cm.^3^], V10_Gy_ − V50_Gy_ [%], Dose_mean_ [Gy] for TBM

Volume [cm^3^], V10_Gy_ − V50_Gy_ [cm.^3^], Dose_mean_ [Gy] for bladder, bowel cavity, all bowel, small bowel, and large bowel

## Discussion

In this work, we propose the Pelvic U-Net architecture for automated and improved pelvic OAR segmentation. Evaluation of our model was performed using observational and multiple quantitative measures.

The Pelvic U-Net resulted in credible and clinically applicable OAR segmentations, which was also demonstrated by the observational and time-based evaluation. This indicates that our model can be used as a supportive tool in the RT treatment planning workflow for anal cancer patients.

Although direct comparisons with other studies should be undertaken with caution, we believe that the results obtained in this work compare favorably with previous studies of deep learning-based OAR segmentations in the pelvic region. In a recent study, Sartor et al. [25] trained a 3D U-Net-like architecture using CT volumes of 191 anorectal cancer patients. The lower mean DSC values in that study compared to our study (e.g., bowel cavity 0.82 vs. 0.95) could be attributed either to differences in model performance or differences in the dataset. Notably, OAR in our study were retrospectively and rigorously contoured on each CT slice by two clinical experts according to clearly defined instructions, in contrast to previous studies, e.g. in the work of Sartor et al. [25] or Men et al. [24], who used clinically available structure sets as the GT. While a retrospective re-contouring is associated with potential drawbacks such as time consumption and a risk of decreased generalizability, the reduced variability of the final structure set most likely facilitates the model performance.

A very limited number of previous studies have used deep learning approaches to differentiate between the small and large bowel. Men et al. [24] trained a 2D dilated CNN, based on a modified VGG-16 model [49], on CT volumes of 278 rectal patients for automatic segmentation of pelvic OAR. Despite the use of oral contrast, the mean DSC values for the small and large bowel (0.62 and 0.65, respectively) were lower than in our study (0.87 and 0.81, respectively).

The overall good segmentation performance of our model was also demonstrated by the observational assessment. None of the pelvic bone marrow, bladder, and bowel cavity structures was rated as not acceptable. Even for the more complex all bowel structure, most segmentations were rated to be either excellent (44%) or good (38%). For small and large bowel, some structures were rated as not acceptable (16% and 27%, respectively). Median ratings between the observers were found to be the same for TBM, bladder and bowel cavity. For small and large bowel, the median ratings varied between 2–3, indicating minor inter-observer variability.

Evaluation of segmentation time presented in this study was performed by comparing model prediction time to manual delineation time, as well as to the time needed to manually correct the model’s predictions. In our study, the mean segmentation time can be reduced from 40 min to about 12 min per patient, including manual correction, resulting in a segmentation time reduction of 70%. In addition, the prediction time of our model was measured to be 4 min for all OAR structures together, reducing manual segmentation time by 90% for cases where no manual intervention is needed. This time might be reduced further with future advances in computational hardware. Segmented CT slices outside of the GT (2 cm above the PTV) were removed in bowel structure predictions. This time was not included in our time-based evaluation but was considered to be negligible.

Important to mention is that not all the presented OAR, e.g., small, and large bowel, are used in today’s clinical routine, due to the time available for manual delineation. The presented Pelvic U-Net model might be a first step to address this issue.

A few outliers for large and small bowel segmentations were found in our test dataset, potentially limiting their clinical use. Nevertheless, compared to other work [24], our model shows improved segmentation quality for these structures. As described earlier, GT segmentations were generated with support from diagnostic CT images with oral contrast enhancement, which helps identifying large and small bowel structures. However, despite the improved image contrast, distinguishing between large and small bowel can be a difficult task even for an experienced radiation oncologist, which might also explain the minor inter-observer variability in the observational ratings. Furthermore, some of the used training patients had a stoma, which might have affected the overall configuration of the bowel structure.

In addition to the observational and quantitative evaluations, we also presented a dosimetric evaluation. A statistically significant difference was found for some of the DVH indices for bladder, all bowel and TBM. For the TBM structure, we hypothesize that the thresholding algorithm, used to assist in manual delineation of the bone marrow, might have led to coarser GT segmentations or even thresholding artefacts (Fig. 5a–e). Further, in some occasional 2D CT slices, tissue near or enclosed by the bone structure was not segmented by the Pelvic U-Net (Fig. 5b, c). Differences between the models’ predictions and the GT might also be caused by a combination of manual delineation errors, e.g., for the bladder structure (Fig. 5f, g), possibly due to the time available for delineation, and errors caused by the deep learning model itself. It should be noted that little is known about the clinical impact of the relatively small observed differences in the evaluated dose parameters. However, one cannot rule out that the differences might have clinical relevance. Therefore, AI-segmentations need to be reviewed and adjusted if needed before clinical use.

Even though our Pelvic U-Net model resulted in improved segmentation metrics and decreases the overall segmentation time, our study comprises certain limitations. First, a single institution dataset was used for both training and testing. Before any firm conclusions can be drawn regarding generalizability, our model needs to be tested on independent datasets. Second, for some OAR there is currently no international consensus on exactly how they should be delineated. For instance, the definition of bowel cavity recommended by the Radiation Therapy Oncology Group (RTOG) [50] differs from the definition used in our study (clearly described in the methods section). If other clinicians and researchers were to use our model, this needs to be considered. To further improve our presented method and the segmentation accuracy for small and large bowel structures, oral contrast CT images could be added to the training data and incorporated in future model training processes.

## Conclusions

We developed and thoroughly evaluated the Pelvic U-Net, a deep-learning model for multi-label segmentation of pelvic OAR structures. The overall segmentation quality was improved, when compared to previous studies. Model predictions resulted in clinically, acceptable, and credible segmentations. Even though manual corrections were needed for some structures, Pelvic U-Net led to average time savings of 70%. We believe that our model can be utilized in the clinical day-to-day planning process for fully automated segmentations for most of the presented OAR. This will enable an accelerated and improved treatment planning process for anal cancer patients treated with external beam radiation therapy.

## Supplementary Information

**Additional file 1**: Detailed evaluation of the 5-fold cross-validation and gradient-weighted Class Activation Mappings (GradCam) obtained from the last convolutional layer of the Pelvic-UNet.

## Data Availability

Due to ethical limitations and institutional regulations, the data used in this study is not shared.

## Abbreviations

ABS: Atlas-based segmentation
BCE: Binary cross-entropy
CNN: Convolutional neural network
CT: Computed tomography
DICOM: Digital Imaging and Communications in Medicine
DSC: Dice similarity coefficient
DVH: Dose-volume histogram
GAP: Global average pooling
GN: Group normalization
GT: Ground truth
HD_95_: Hausdorff distance (95th percentile)
IBM: Iliac bone marrow
LPBM: Lower pelvic bone marrow
LSBM: Lumbosacral bone marrow
MSD: Mean surface distance
OAR: Organs at risk
ReLU: Rectified linear unit
RT: Radiation therapy
RTOG: Radiation Therapy Oncology Group
SA: Shuffle attention
TBM: Total bone marrow
TPS: Treatment planning system
