# Supplementary material for: Pelvic U-Net: multi-label semantic segmentation of pelvic organs at risk for radiation therapy anal cancer patients using a deeply supervised shuffle attention convolutional neural network
Source: Radiat Oncol. 2022 Jun 28;17:114. doi: 10.1186/s13014-022-02088-1 (PMC9238000; doi:10.1186/s13014-022-02088-1)
Supplement: Supplementary file 1 — Additional file 1: Detailed evaluation of the 5-fold cross-validation and gradient-weighted Class Activation Mappings (GradCam) obtained from the last convolutional layer of the Pelvic-UNet. [file 13014_2022_2088_MOESM1_ESM.docx]

**Supplementary material**

**Table S1.** Mean dice similarity coefficient (DSC) computed over the validation dataset for each fold of the 5-fold cross-validation.

| **Dice similarity coefficient 5-fold cross-validation** $\left( \bar{\boldsymbol{x}}\boldsymbol{\pm sd} \right)$ | | | | | | |
| --- | --- | --- | --- | --- | --- | --- |
|  | Fold 0  (n = 29) | Fold 1 (n = 29) | Fold 2 (n = 29) | Fold 3 (n = 28) | Fold 4 (n = 28) |  |
| **TBM** | 0.97 ± 0.01 | 0.97 ± 0.01 | 0.96 ± 0.01 | 0.97 ± 0.01 | 0.97 ± 0.00 |  |
| **LPBM** | 0.96 ± 0.01 | 0.96 ± 0.01 | 0.94 ± 0.02 | 0.95 ± 0.01 | 0.95 ± 0.01 |  |
| **IBM** | 0.95 ± 0.01 | 0.95 ± 0.01 | 0.93 ± 0.02 | 0.95 ± 0.01 | 0.95 ± 0.01 |  |
| **LBM** | 0.95 ± 0.02 | 0.95 ± 0.02 | 0.94 ± 0.02 | 0.95 ± 0.02 | 0.95 ± 0.01 |  |
| **Bowel cavity** | 0.94 ± 0.03 | 0.94 ± 0.03 | 0.95 ± 0.02 | 0.94 ± 0.02 | 0.94 ± 0.01 |  |
| **All bowel** | 0.90 ± 0.02 | 0.90 ± 0.02 | 0.90 ± 0.02 | 0.90 ± 0.01 | 0.90 ± 0.02 |  |
| **Small bowel** | 0.86 ± 0.08 | 0.86 ± 0.08 | 0.82 ± 0.11 | 0.83 ± 0.07 | 0.87 ± 0.03 |  |
| **Large bowel** | 0.86 ± 0.06 | 0.86 ± 0.06 | 0.77 ± 0.11 | 0.81 ± 0.09 | 0.81 ± 0.10 |  |
| **Rectum** | 0.88 ± 0.06 | 0.88 ± 0.06 | 0.85 ± 0.07 | 0.87 ± 0.07 | 0.88 ± 0.05 |  |
| **Bladder** | 0.93 ± 0.08 | 0.93 ± 0.08 | 0.91 ± 0.08 | 0.92 ± 0.05 | 0.93 ± 0.05 |  |

**Table S2.** Mean 95^th^ percentile of the Hausdorff distance (HD_95_) computed over the validation dataset for each fold of the 5-fold cross-validation.

| **HD_95_ 5-fold cross-validation** $\left( \bar{\boldsymbol{x}}\boldsymbol{\pm sd} \right)$ | | | | | | |
| --- | --- | --- | --- | --- | --- | --- |
|  | Fold 0  (n = 29) | Fold 1 (n = 29) | Fold 2 (n = 29) | Fold 3 (n = 28) | Fold 4 (n = 28) |  |
| **TBM** | 2.19 ± 1.04 | 2.38 ± 0.69 | 2.49 ± 0.74 | 2.21 ± 0.82 | 2.35 ± 0.82 |  |
| **LPBM** | 3.94 ± 1.72 | 4.28 ± 1.57 | 4.70 ± 2.52 | 3.72 ± 1.70 | 3.62 ± 1.75 |  |
| **IBM** | 3.50 ± 1.52 | 3.61 ± 1.43 | 4.47 ± 2.64 | 3.54 ± 1.68 | 3.45 ± 1.92 |  |
| **LBM** | 3.96 ± 4.49 | 3.34 ± 1.40 | 3.29 ± 0.97 | 3.76 ± 2.57 | 3.21 ± 0.94 |  |
| **Bowel cavity** | 6.04 ± 3.77 | 4.63 ± 1.78 | 5.05 ± 3.26 | 9.65 ± 2.17 | 5.16 ± 2.53 |  |
| **All bowel** | 3.38 ± 1.55 | 3.44 ± 1.09 | 3.90 ± 3.01 | 3.61± 1.25 | 3.08 ± 0.52 |  |
| **Small bowel** | 10.70 ± 24.08 | 7.82 ± 6.67 | 10.14 ± 14.98 | 8.64 ± 7.75 | 5.23 ± 3.71 |  |
| **Large bowel** | 8.13 ± 9.34 | 17.35 ± 15.24 | 25.64 ± 26.98 | 18.04 ± 19.69 | 13.41 ± 13.47 |  |
| **Rectum** | 5.57 ± 3.49 | 9.98 ± 8.48 | 6.85 ± 4.36 | 8.09 ± 7.02 | 9.30 ± 11.83 |  |
| **Bladder** | 4.11± 4.94 | 4.90 ± 10.40 | 4.14 ± 3.01 | 3.46 ± 1.87 | 3.50 ± 1.55 |  |


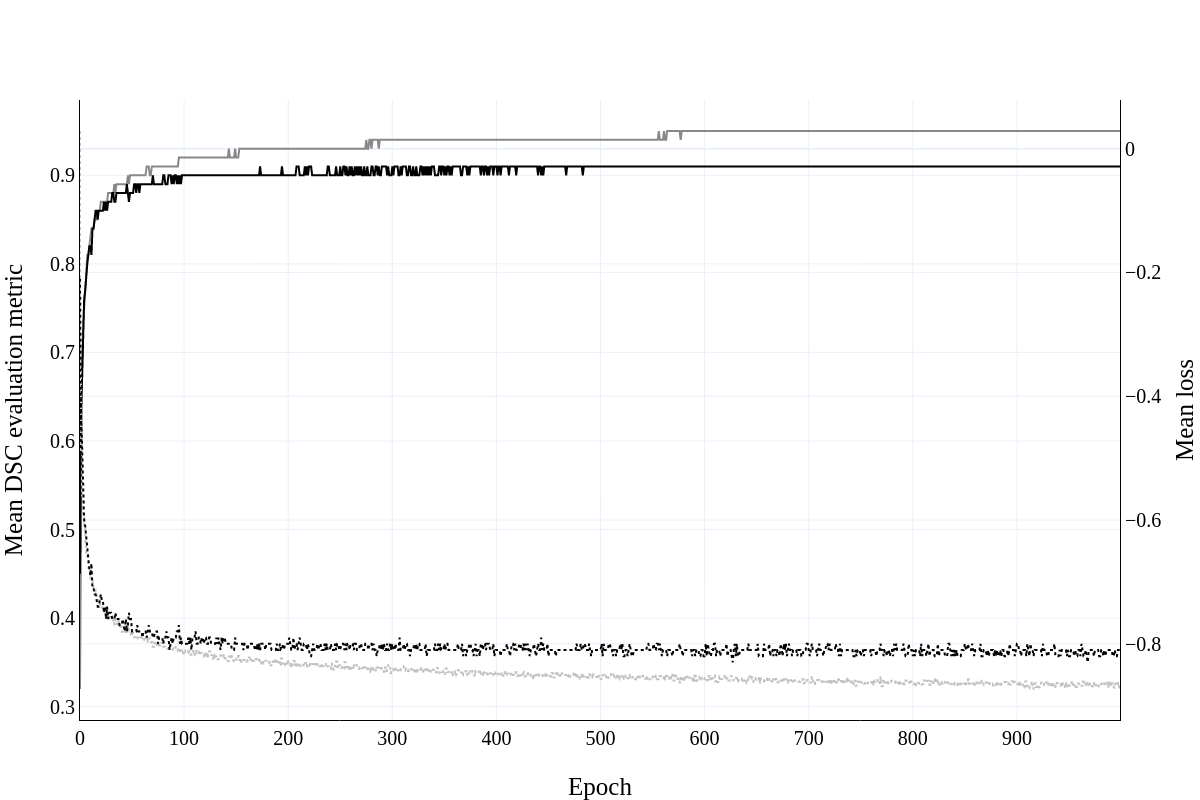


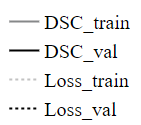


**Figure E1**. Mean dice similarity coefficient (DSC) evaluation metric and mean loss as a function of epochs. Mean loss and mean DSC were computed over all 5 cross-validation models and are shown for both, the training, and the validation data.


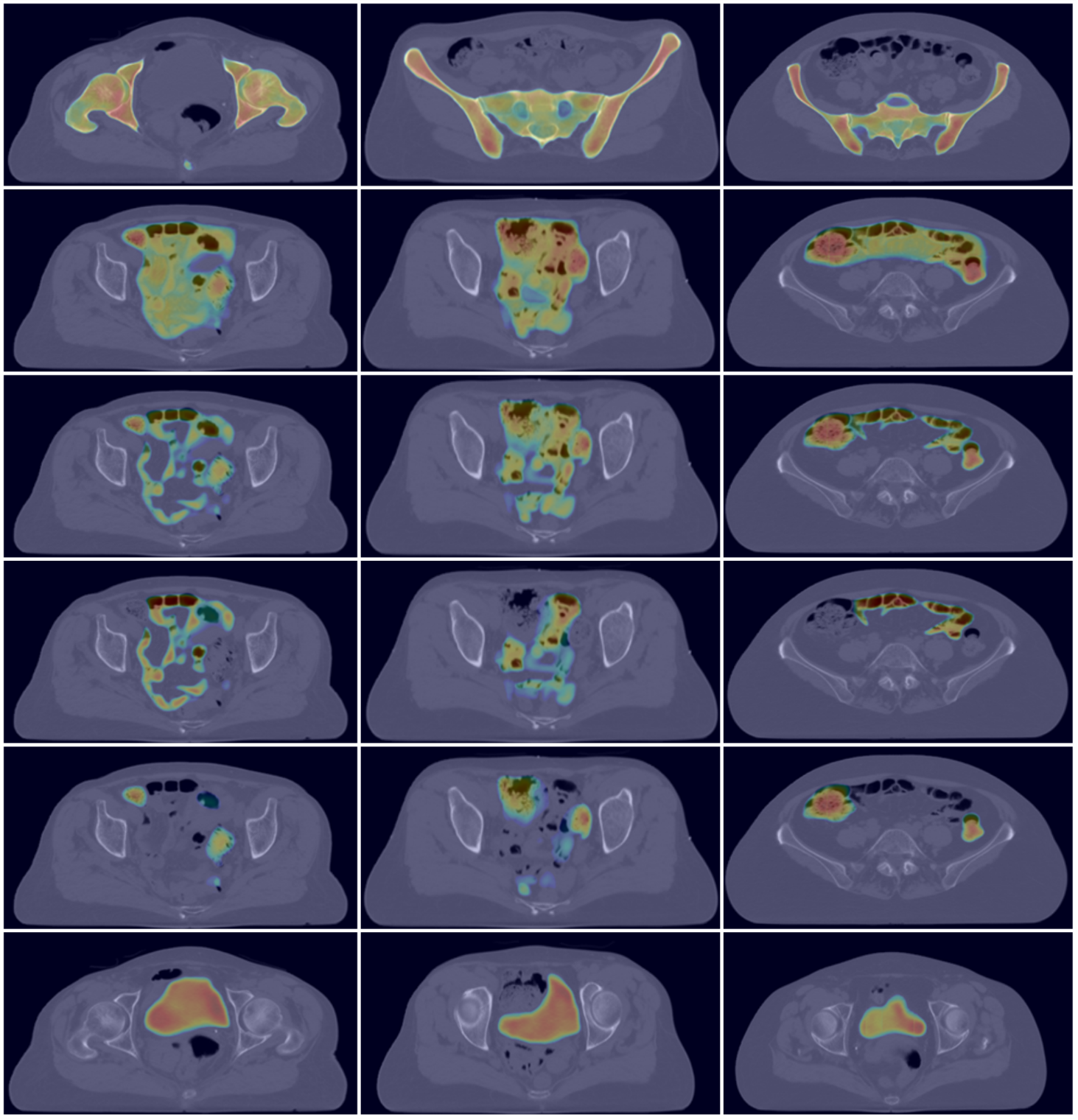


**Test patient 1**

**Test patient 2**

**Test patient 3**

**TBM**

**Bowel cavity**

**All bowel**

**Small bowel**

**Large bowel**

**Bladder**

**Figure E2**. Gradient-weighted Class Activation Mappings (GradCam) **[1]** derived for three patients from the test dataset. All GradCams are shown in the axial plane for multiple organs at risk (OAR) and were derived from the last convolutional layer of the Pelvic U-Net. Class activations were normalized between 0 (blue) – 1 (red), with 1 indicating the highest activation.

**References**

**[1]** R. R. Selvaraju, M. Cogswell, A. Das, R. Vedantam, D. Parikh and D. Batra, "Grad-CAM: Visual Explanations from Deep Networks via Gradient-Based Localization," 2017 IEEE International Conference on Computer Vision (ICCV), 2017, pp. 618-626, doi: 10.1109/ICCV.2017.74.
